# Characteristics of soil C:N:P stoichiometry and enzyme activities in different grassland types in Qilian Mountain nature reserve-Tibetan Plateau

**DOI:** 10.1371/journal.pone.0271399

**Published:** 2022-07-14

**Authors:** Qiang Li, Junyin Yang, Guoxing He, Xiaoni Liu, Degang Zhang

**Affiliations:** Gansu Agricultural University/Key Laboratory of Grassland Ecosystem of the Ministry of Education, College of Grassland Science, Lanzhou, China; Tennessee State University, UNITED STATES

## Abstract

This research was designed to explore the variation characteristics of soil C:N:P stoichiometry and enzyme activity in the Qilian Mountains different grassland types. Thus, 7 grassland types (Upland meadow: UM, Alpine meadow: AM, Temperate steppe: ST, Alpine steppe: AS, Temperate Desert Steppe: TDS, Temperate Desert: TD, Alpine desert: AD) of Qilian Natural Reserve were selected to analyze the variation characteristics of soil enzyme activities and stoichiometry of different grassland types and its relationship with environmental factors. The study indicated that the C/N, C/P, and N/P of different grasslands ranged from 5.08 to 17.35, 2.50 to 72.29, and 0.53 to 4.02.The ranking of different types grassland for the C/N was TS ≥ AM ≥ UM ≥ AS ≥ TDS > AD > TD, and the changing pattern of C/P and N/P is similar to that of C/N. The ranking of different types grassland for the urease enzyme activity was UM ≈AS > AD ≈TDS ≈TS ≈AM > TD, and TS ≈AM ≈UM ≈AS ≈AD > TDS > TD for alkaline phosphatase enzyme activity, and AS ≈AM ≈TS ≈TDS≥UM ≥TD ≈AD for catalase enzyme activity. Based on N/P ratio and RDA analysis, nitrogen was the main factor limiting the grassland productivity, and pH, TN, SOC, Richness index and Simpson diversity index were the main environmental factors affecting the soil C:N:P stoichiometry and enzyme activities. Cluster analysis showed that 7 grassland types were clustered into three categories. In conclusion, the stoichiometric characteristics and soil enzyme activities of different grasslands vary with grassland types. Nitrogen was the main factor limiting the grasslands productivity, and pH, TN, SOC, Richness index and Simpson diversity index were the main environmental factors affecting the soil C:N:P stoichiometry and enzyme activities, and the grassland Qilian Mountain can be managed in the ecological district according to the clustering results. The results of this study can provide data support and theoretical guidance for the scientific management and ecological protection of grassland in Qilian Mountains Reserve.

## Introduction

Ecological chemometrics is a theoretical science that explores the regular changes in the ratios of chemical elements (mainly carbon, nitrogen, phosphorus, and potassium) caused by the disturbance of external factors that affect the conservation of energy flow and material cycles in ecosystems, and provides a simpler way to research the mechanism of interexistence between chemical elements and energy cycle of biological substances [1,2]. Carbon (C), nitrogen (N), phosphorus (P) and potassium (K) are the macronutrients of the soil and the main constituent elements of the plant organism. The decomposition of dead branches and leaves returns some of the nutrients to the soil, which continues to provide the necessary nutrients for healthy plant growth [3–5]. Soil, as an indispensable component of grassland ecosystems, is also the place where vegetation communities survive and develop, and the succession process of vegetation communities in turn affects the soil development process, and the two are dependent on each other and mutually constrained [6–8]. Therefore, the influence of soil ecological chemometric characteristics on nutrient cycling and ecosystem balance in grasslands cannot be ignored. Soil enzymes are mainly derived from residues of microorganisms, animals, plants and secretions from their metabolic processes [9,10], actively participating in the biochemical process of the soil system and a key link of "plant-soil enzyme-soil nutrients" [11]. As important indicators of soil fertility evaluation, soil enzyme activity and soil nutrients play an important role in material circulation and energy transformation of soil ecosystems [12], indirectly affecting the circulation of carbon, nitrogen, phosphorus and other elements in the soil. In the context of global climate change, soil enzymes are increasingly critical in the ecologically fragile Qilian Mountain grassland ecosystem [9]. Studies found that [13–15] soil enzymes in various vegetation and grassland have different sensitivity to water and heat, while the sensitivity of the same soil enzyme was also different under vegetation types. Therefore, the influence of soil enzyme characteristics on nutrient cycling and ecosystem balance in grasslands cannot be ignored.

Qilian Mountain is located on the eastern edge of the Tibet Plateau in China, adjacent to the Mongolian Plateau and the Loess Plateau [16]. It is the birthplace of the inland rivers for Heihe, Shiyang and Shule, the water source of the Hexi Oasis in northwest China [17], and also one of the most sensitive regions for global climate change [17–19]. Due to altitude and region differences, Qilian Mountains boasts many types of grassland and the grasslands was the largest vegetation type in this region [20]. The study found that, changes in grassland types could cause changes in many natural factors and ecological processes [21], such as soil enzyme activity, soil nutrients [22–25], and vegetation characteristics [26,27]. At present, what are the changing patterns of soil ecological chemometric characteristics and soil enzyme activity of different grassland types in Qilian Mountain? And there is no relevant explanation for this scientific question. Scholars had conducted more studies on Qilian Mountain grasslands, but these studies have mainly focused on vegetation characteristics, biodiversity and soil nutrients [6,7,13–15,28], and no studies have been reported on soil ecological chemometrics and soil enzyme activities in different grassland types. This paper selected the Qilian Mountain grassland as the research object, to identified the characteristics changes of soil stoichiometric and enzyme activity in different grassland types. To explore the following questions: 1) Are there nutrient limitations in different grassland types in the Qilian Mountains? 2) What are the environmental factors that affect grassland soil stoichiometric characteristics and soil enzyme activities? 3) Is the Qilian Mountains grassland managed in different zones according to the grassland types? The solution of the above scientific problems will further reveal the relationship between the nutrient cycling mechanism and ecosystem balance in the Qilian Mountains, and provide a basis for the protection and scientific management of the local grassland ecosystem.

## Materials and methods

### Study area

The study sites were located in the Qilian Mountains Nature Reserve of eastern Qinghai-Tibetan Plateau, China (94°10′-103°04′E, 35°50′-39°19′N) (http://www.qilianshan.com.cn/html/1/271/160/168/index.html). At the horizontal direction, four vegetation zones existed in the order of forest, shrub, grassland and desert from southeast to northwest. At the vertical direction, three vegetation belts are distributed as steppe, forest and alpine meadow from low to high altitude (856–5564 m). The main soil types are aridisols, inceptisols and entisols. The precipitation varies from 100 to 500 mm, from June to September. The average annual temperature is approximately -2.0°C; the average annual relative humidity range is 20%-70%; the annual evaporation is 1200–1400 mm; the frost-free period is 90–120 days [20].

### Sample selection

This research area was mainly focused on the Qilian mountain nature reserve in Gansu Province, China. The grassland types and utilization were showed in Table 1. Plant species in the sample field were identified and classified by the grassland College of Gansu Agricultural University. Although the voucher specimen of plant species has not been deposited in a publicly available herbarium, plant species can be investigated in the field. Meanwhile, the plant and soil samples were collected with permission from the Qilian Mountains Nature Reserve Administration, Gansu province, China. All authors committed that all methods were carried out in accordance with relevant guidelines and regulations.

**Table 1:** Basic information of the sample plots.

Type grassland	Altitude m	longitude and latitude	Main plant species	Species
Upland meadow (UM)	3114	37°11′36.47″N 102°43′42.73″E	*Potentilla anserina L*., *Poa annua L*., *Elymus nutans Griseb*., *Melissilus ruthenicus(L*.*) Peschkova*, *Artemisia annua Linn*.	14
Alpine meadow (AM)	2977	37°10′48.66″N 102°47′13.83″E	*Polygonum viviparum L*., *Kobresia myosuroides (Villars) Fiori*, *Melissilus ruthenicus(L*.*) Peschkova*, *Artemisia annua Linn*., *Saussurea japonica DC*.	15
Temperate steppe (ST)	2817	37°22′13.68″N 102°40′44.93″E	*Poa annua L*., *Kobresia myosuroides (Villars) Fiori*, *Stipa capillata Linn*., *Potentilla anserina L*., *Artemisia annua Linn*.	20
Alpine steppe (AS)	3735	39°16′32.99″N 97°42′52.57″E	*Stipa purpurea*, *Kobresia myosuroides (Villars) Fiori*, *Poa annua L*., *Potentilla anserina L*., *Androsace umbellata*	16
Temperate Desert Steppe (TDS)	2139	38°57′57.23″N 99°47′41.95″E	*Sympegma regelii Bunge*, *Salsola collina Pall*., *Allium polyrhizum Turcz*, *Stipa capillata Linn*., *Ajania nematoloba*	6
Temperate Desert (TD)	1358	39°29′29.11″N 99°18′45.00″E	*Nitraria tangutorum Bobr*, *Nitraria sphaerocarpa Maxim*, *Suaeda glauca (Bunge) Bunge*, *Sympegma regelii Bunge*	4
Alpine desert (AD)	4290	39°15′34.39″N 97°45′6.70″E	*Rhodiola rosea L*., *Saussurea japonica DC*., *Kobresia myosuroides (Villars) Fiori*	6

### Sample collection

The sampling time was from July to August 2019, when the plants were in full bloom. The central area of the typical distribution area of the above 7 grassland types (Upland meadow: UM, Alpine meadow: AM, Temperate steppe: ST, Alpine steppe: AS, Temperate Desert Steppe: TDS, Temperate Desert: TD, Alpine desert: AD) were selected for the sampling sites (Table 1). A total of 6 random sampling quadrates (1 m × 1 m) were selected in each site. In each quadrate, plant species, coverage, height, the density of the respective species and aboveground biomass were measured and recorded. Aboveground parts of the green plants of the respective species were harvested by clipping to the soil surface. All the aboveground plant samples were placed into envelopes and then tagged, respectively. All the green plant samples were immediately dried at 105°C for 0.5 h, then oven-dried at 60°C for 48 h and weighed [20].

Meanwhile, a 60-meter sample line was set for each sample site. The sample spots were set at a 20-meter interval. Four soil samples were taken around each sample spot using soil drills with a depth of 0–30 cm, respectively. Four soil samples from each layer were mixed as one sample. The samples were put into a sample bag and taken back indoors for air-drying, measured for soil organic matter, Total N, Total P, pH and soil enzyme activity.

### Sample determination

Soil samples were air-dried at room temperature, where visible roots and other debris were removed. Each composite soil sample was sieved through a 2-mm sieve. The Walkley-Black method was used to determine soil organic matter [29]. the Kjeldahl acid digestion method was used to determine total N (Foss Kjeltec 8400, FOSS, DK) [29]. The Mo-Sb colorimetry (UV-2102C, UNICO, Shanghai, China) was used to measure the total P [29]. The potential method (water/soil ratio: 2.5: 1) was used to measure soil pH value. the phenol sodium-hypochlorite sodium colorimetric method was used to measure urease enzyme activity [30]. According the amount of glucose (mg) generated in a 1-g soil sample after cultivation at 37°C for 24 h was calculated Sucrase enzyme activity [30]. The disodium phenyl phosphate method was used to measure alkaline phosphatase enzyme activity [30]. The KMnO_4_ titration was used to measure catalase enzyme activity [30].

### Statistical analyses

Data statistics and plotting were carried out by Excel 19.0. All results were presented as mean and standard deviations. One-way ANOVA (P < 0.05), Correlation and cluster analysis were performed with SPSS version 19.0 (SPSS Inc., Chicago, IL, USA). PCA analysis was performed using Canoco 5.0.

The IV (Important Values) of each plant species was calculated by the following formula [14,31]:

$$IV=\frac{relative\;coverage+relative\;height+relative\;density+relative\;dry\;weight}{4}$$ (1)

Plant diversity was estimated using the three standard multi-dimensional biodiversity indices, i.e., Pielou Evenness index, Shannon-Weiner (H’). Simpson diversity index was calculated based on the following equations.

$$H\prime =-\sum_{i=1}^{s}P_{i}\ln\;P_{I}$$ (2)

where H’ represents the Shannon-Weiner index; P_i_, the total number of individual species proportion of ith species in the community; S, the encountered species number; P_i_, the proportion of the total number of individual species belonging to I th species in the community; ln P_i_, the natural logarithm of P_i_.

$$Simpson\;diversity\;index=1‐\sum_{i=1}^{S}P_{i}{}^{2}$$ (3)

where S represents encountered species number; Pi, the total number of individual species proportion of i th species in the community.

PielouEvennessindex=H′/lnS (4)

where H’ represents the Shannon-Weiner index; S, the number of species

Richnessindex=S (5)

where S means the number of species.

## Result

### Vegetation characteristics and soil nutrient

**Fig 1:**
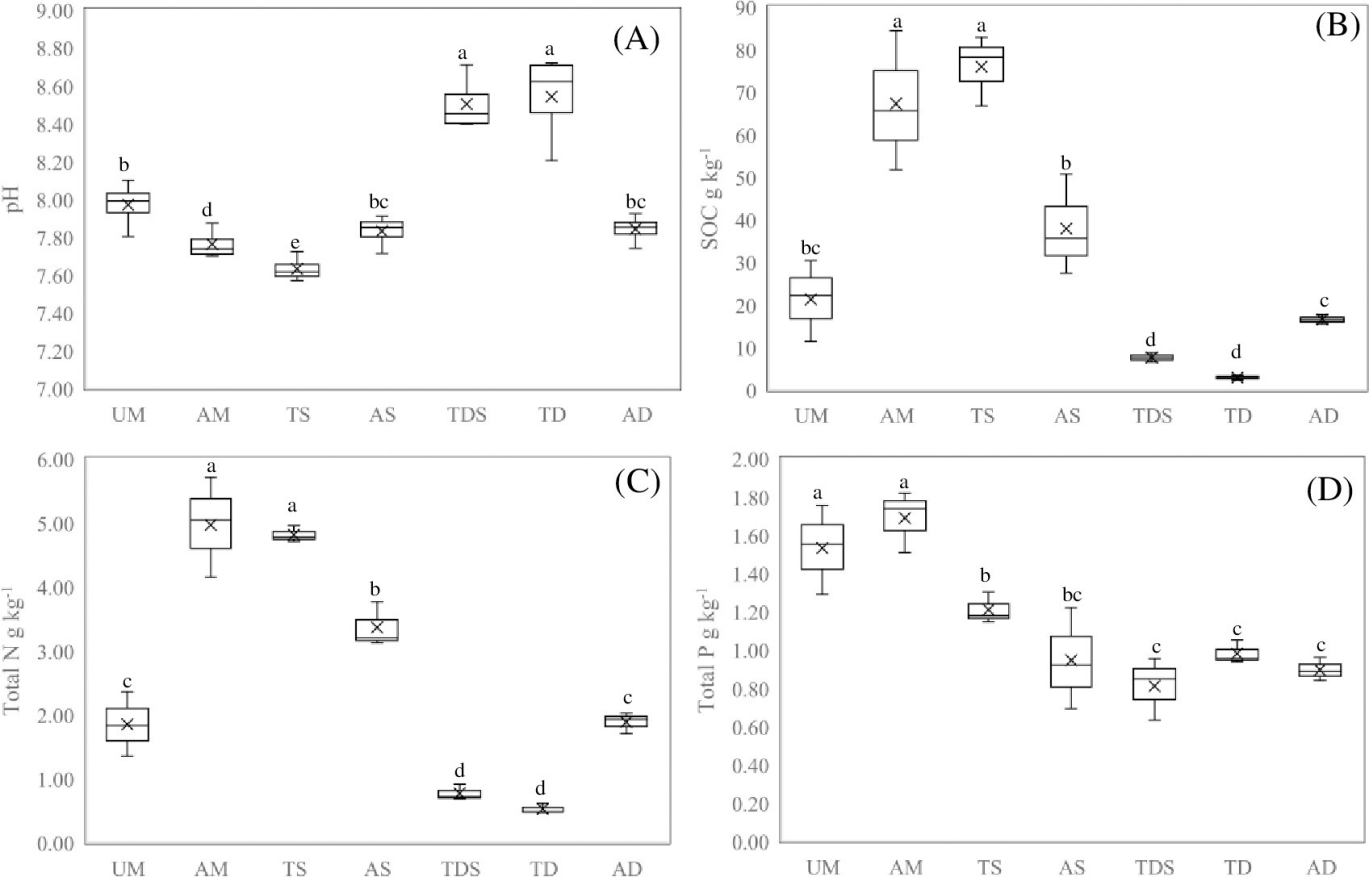
Content of C, N, P and pH in different types grassland. Different lower-case letters mean different type grasslands significant differences at 0.05 level. TS, Temperate steppe; AM, Alpine meadow; AS, Alpine steppe; UM, Upland meadow; AD, Alpine desert; TDS, Temperate Desert Steppe; TD, Temperate Desert.

Total coverage of different grassland types ranged from 28.33% to 85.00% (Table 2), the ranking of different types grassland for was AM ≈ UM ≈TS ≈ AS> TDS >TD ≈ AD. And grass layer height of different grassland types ranged from 5.63 to 27.20 cm (Table 2), the ranking of different types grassland for was TD ≈ AD > TS ≈ AS ≈ UM > AM >TDS. And AGB of different grassland types ranged from 136 to 486 g·m^-2^ (Table 2), the ranking of different types grassland for was TS ≈ AS ≈ UM > TD ≈ AM >TDS > AD. The variation pattern of Shannon Weiner diversity index in different grassland types was similar to that of AGB. And Pielou Evenness index of different grassland types ranged from 0.92 to 0.99 (Table 2), the ranking of different types grassland for was TS ≈ AS ≈ UM > TD ≈ AM >TDS > AD. Pielou Evenness index of different grasslands ranged from 0.92 to 0.99, the Pielou Evenness index in AD was higher than that in AS, in AS was higher than that in AD, but no significant differences were found between other treatments. Simpson diversity index of different grasslands ranged from 0.09 to 0.31, the ranking of different types grassland for was TD > TDS ≈ AD > TS ≈ AS ≈ UM ≈AM. Richness index of different grasslands ranged from 3.52 to 9.24, the ranking of different types grassland for was TS ≈ AS > UM ≈AM > TD ≈ TDS ≈ AD. The pH of different grasslands ranges from 7.63 to 8.54(Fig 1A), the pH in TD and TDS was higher than that in UM, AS, and AD, and in UM, AS and AD was higher than that in AM, but no significant differences were found between other treatments. The SOC of different grasslands ranged from 2.87 to 75.93 g kg^-1^(Fig 1B), The ranking of different types grassland f was TS ≈ AM > AS > AD > TDS ≈TD. The variation pattern of total N in different grassland types was similar to that of SOC (Fig 1C).

**Table 2:** Vegetation characteristics of different type grasslands.

Item	Total Coverage %	Grass layer height cm	Aboveground biomass (AGB) g m^-2^	Shannon-Weiner index	Pielou Evenness index	Simpson diversity index	Richness index
UM	81.67 ±2.89a	19.57 ±1.86b	415 ±36a	2.55 ±0.19a	0.96 ±0.01ab	0.09 ±0.01c	6.61 ±0.42b
AM	85.00 ±5.00a	8.28 ±0.17c	368 ±13b	2.59 ±0.25a	0.96 ±0.01ab	0.08 ±0.03c	7.02 ±0.53b
TS	80.00 ±5.00a	19.13 ±2.57b	486 ±20a	2.85 ±0.63a	0.95 ±0.00ab	0.07 ±0.01c	9.24 ±0.68a
AS	80.00 ±2.00a	16.17 ±3.86b	441±43a	2.60 ±0.38a	0.94 ±0.01b	0.09 ±0.04c	8.27 ±0.45a
TDS	43.75 ±2.07b	27.20 ±4.79a	246 ±26c	1.74 ±0.16b	0.97 ±0.02ab	0.18 ±0.06b	3.52 ±0.26c
TD	31.67 ±2.88c	26.51 ±3.88a	329 ±40b	1.27 ±0.02c	0.92 ±0.01c	0.31 ±0.07a	3.81 ±0.71c
AD	28.33 ±2.88c	5.63 ±1.33d	136 ±6.4d	1.77 ±0.11b	0.99 ±0.02a	0.17 ±0.03b	4.01 ±0.36c

Note: Data are presented as the mean ±SD; Different small letters in the same row mean significant difference at 0.05 level. TS, Temperate steppe; AM, Alpine meadow; AS, Alpine steppe; UM, Upland meadow; AD, Alpine desert; TDS, Temperate Desert Steppe; TD, Temperate Desert. SD, Standard deviation; CV: Coefficient of Variation; AGB: Aboveground biomass.

The total P of different grasslands ranges from 1.03 to 1.79 g kg^-1^(Fig 1D), The ranking of different types grassland was AM ≈ UM > TS ≈ AS > TD ≈ AD ≈ TDS.

### Stoichiometric ratio of C, N, and P

**Fig 2:**
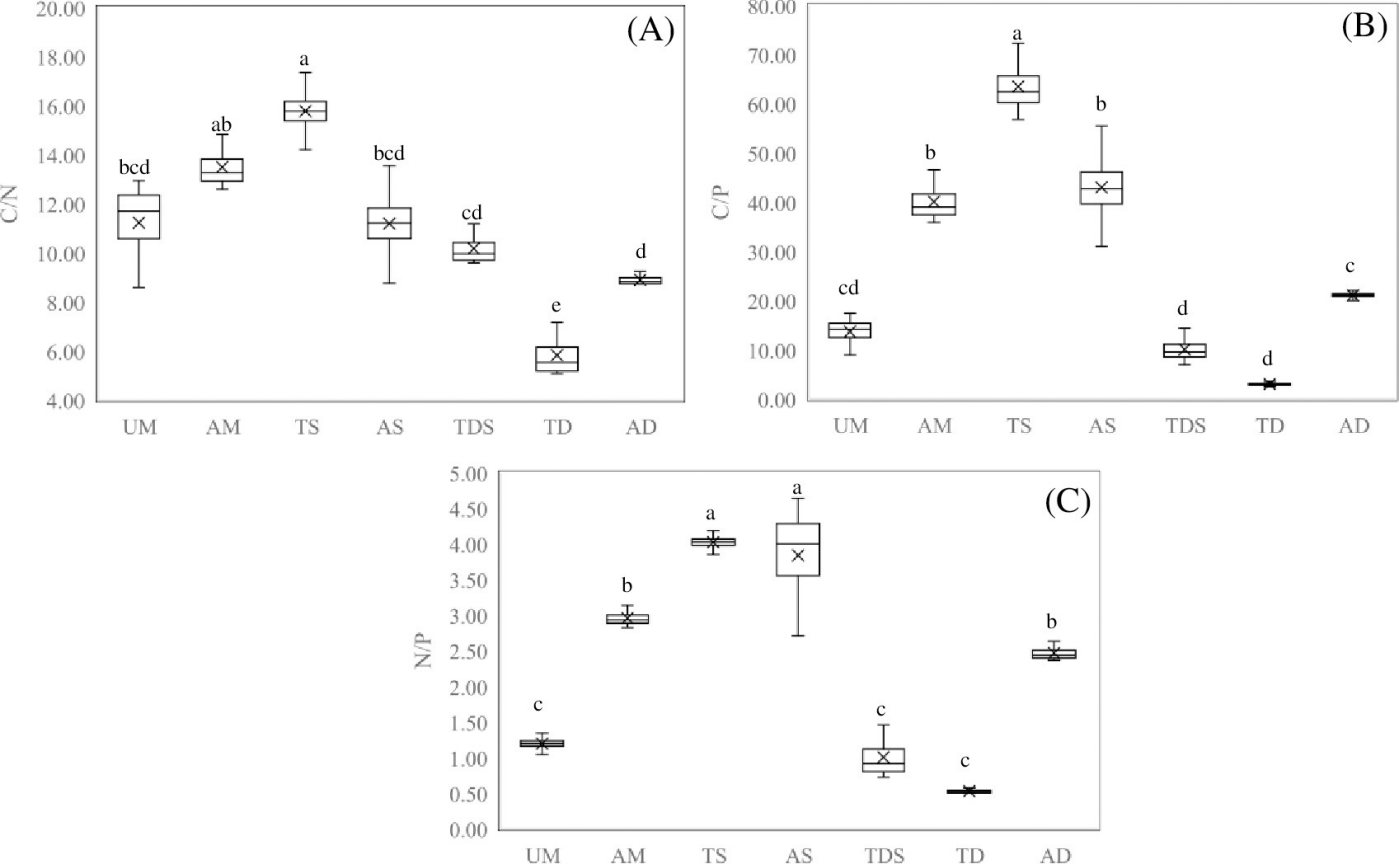
Stoichiometric ratio of C, N and P in different types grassland.

The C/N of different grasslands ranged from 5.08 to 17.35 (Fig 2A). C/N in TS was higher than that in UM and AS, and in AM was higher than that in TDS, and in AD, UM, and AS is higher than that in TD, but no significant differences were found between other treatments. The C/P of different grasslands ranged from 2.50 to 72.29 (Fig 2B). The N/P of different grasslands ranges from 0.53 to 4.02 (Fig 2C). The changing pattern of C/P and N/P is similar to that of C/N. N/P ratio can be used as an index for determining the nutrient factors that limit productivity, and N/P<10 and N/P > 20 are used as indicators to evaluate the productivity of vegetation limited by nitrogen or phosphorus (Li et al., 2018). The N/P of different grasslands ranged from 0.53 to 4.04, while the plants’ productivity of different grassland was mainly limited by nitrogen.

### Soil enzyme activity

**Fig 3:**
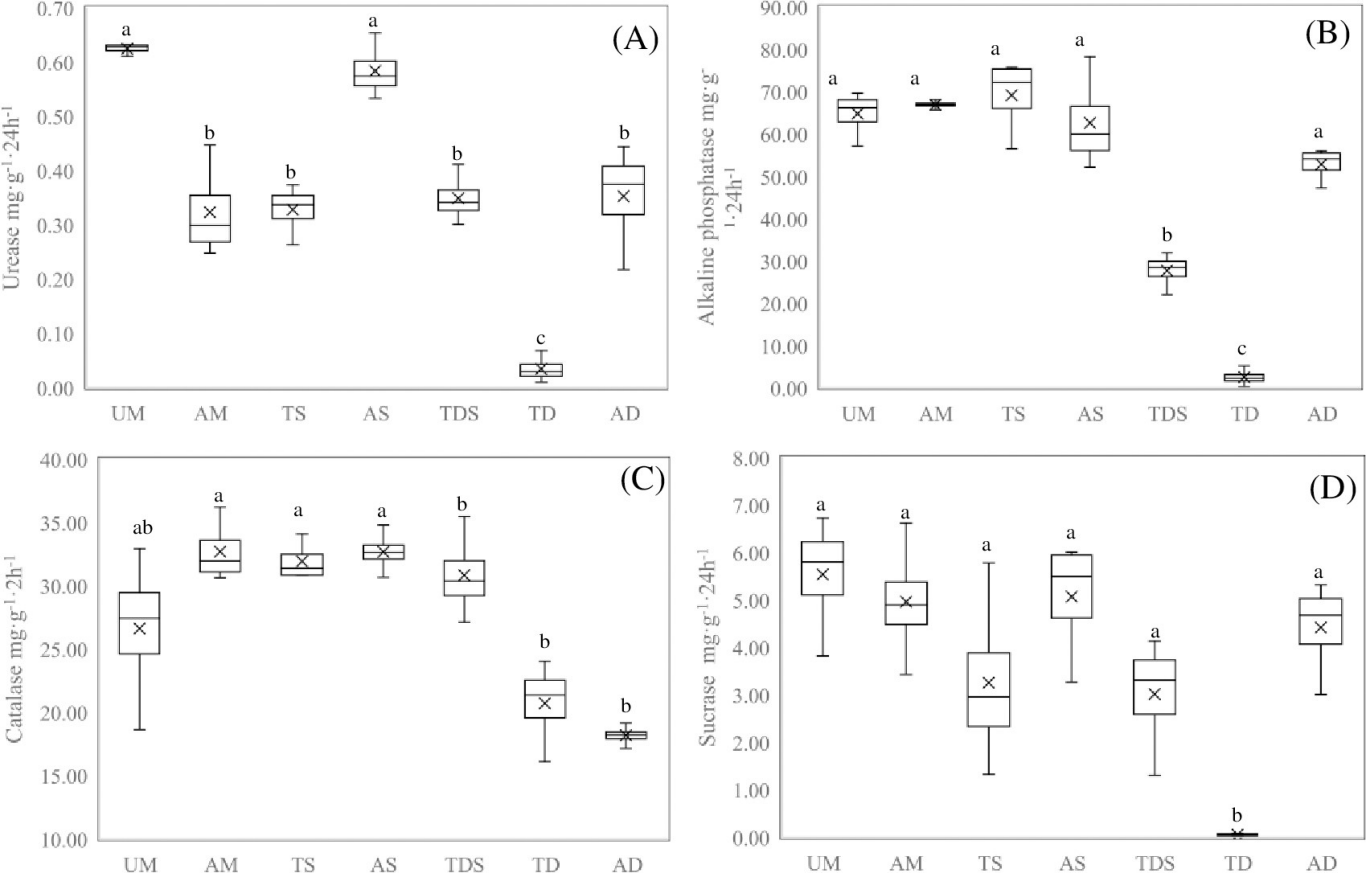
Soil enzyme activity in different types grassland.

Fig 3A shows that the urease enzyme activity of different grasslands is from 0.03 to 0.62 mg·g^-1^·24h^-1^. Urease enzyme activity in UM and AS was higher than that in AM, TS, TDS, and AD, and was higher than that in TD, but no significant differences were found between other treatments. The ranking of different types grassland for the urease enzyme activity was UM ≈ AS > AD ≈ TDS ≈ TS ≈ AM > TD. Alkaline phosphatase enzyme activity of different grasslands ranged from 2.46 to 69.09 mg·g^-1^·24h^-1^ (Fig 3B). The enzyme activity of alkaline phosphatase in UM, AM, TS, AD, and AS was higher than that in TDS, and in TDS was higher than that in TD, but no significant differences were found between other treatments. Therefore, the alkaline phosphatase enzyme activity was in a ranking order of TS ≈ AM ≈ UM ≈ AS ≈ AD > TDS > TD. Catalase enzyme activity of different grasslands ranges from 2.46 to 69.09 mg·g^-1^·24h^-1^ (Fig 3C). The catalase enzyme activity in AM, TS, and AS was higher than that in AD, but no significant differences were found between other treatments. Therefore, catalase enzyme activity was in a ranking order of AS ≈ AM ≈ TS ≈ TDS≥ UM ≥ TD ≈ AD. Sucrase enzyme activity of different grasslands ranged from 0.03 to 2.29 mg·g^-1^·24h^-1^ (Fig 3D). Sucrase enzyme activity in TD was lower than that in UM, AM, TS, AD, TDS, and AS, but no significant differences were found between other treatments.

### Relationship between soil stoichiometric, enzyme activity and environmental factors

**Fig 4:**
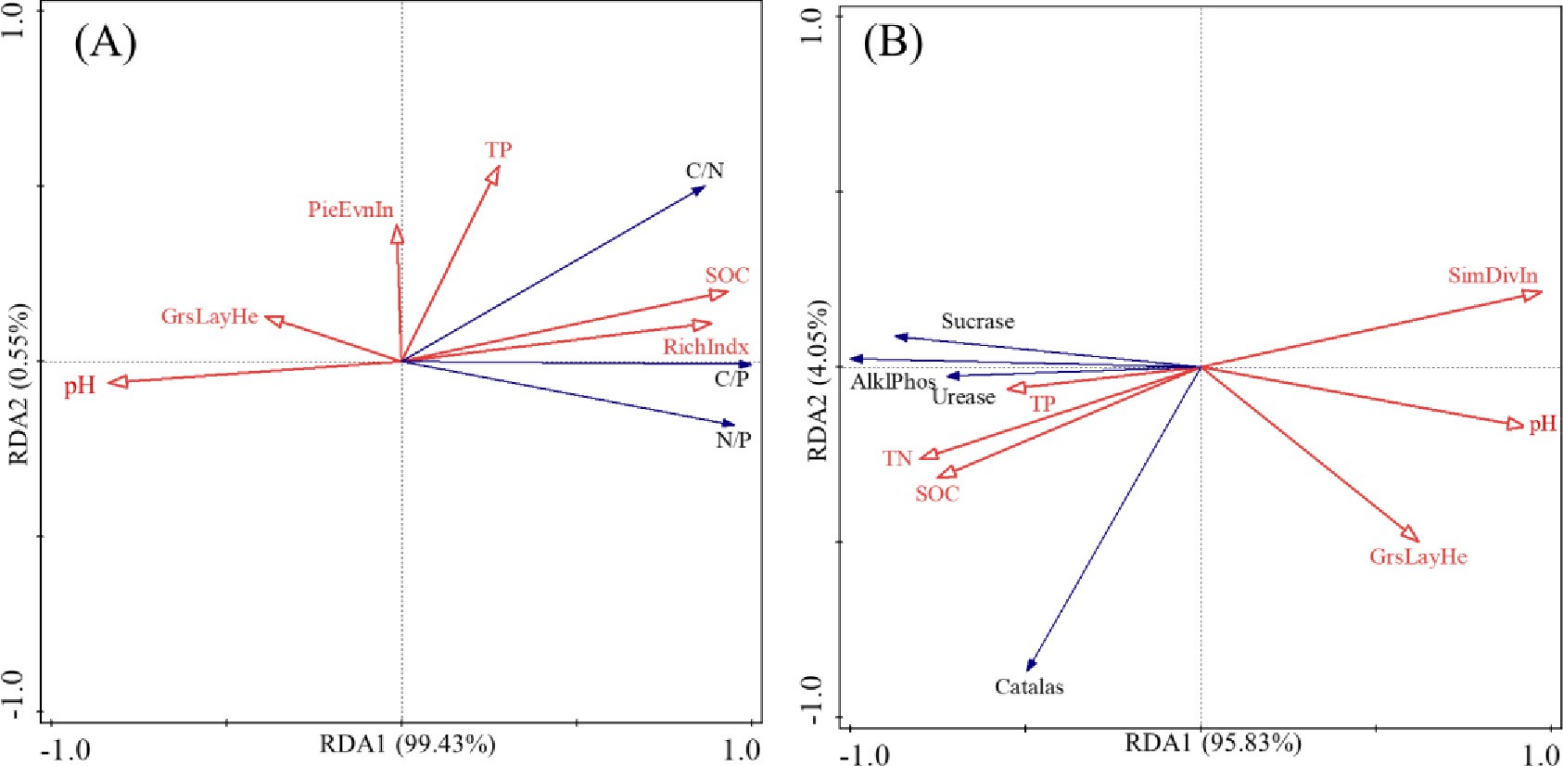
RDA analysis of soil stoichiometric (A) and enzyme activity (B) with environmental factors. Note: Alklphos: Alkaline phosphatase; SimDivIn: Simpson diversity index; Grsslayhe: Grass layer height; PieEvin: Pielou Evenness index; Richindex: Richness index.

**Fig 5:**
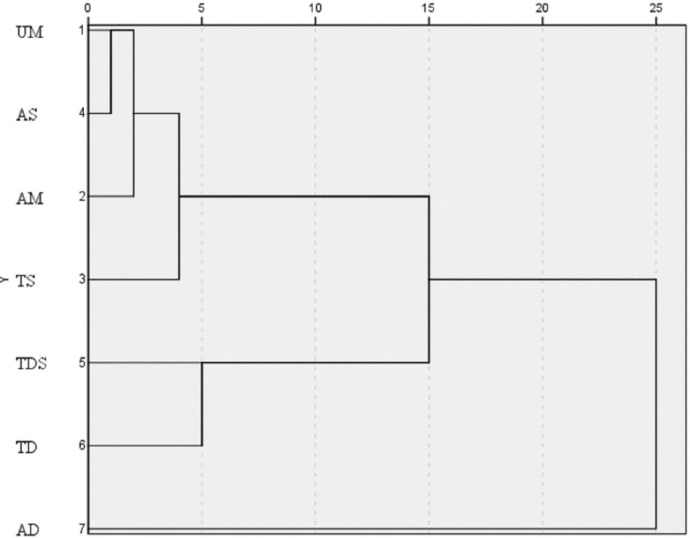
Clustering analysis of different types grassland.

The correlations between soil stoichiometric, enzyme activity and environmental factors are shown in Table 3. There were significantly correlations between C/N and total coverage, Shannon-Weiner, Simpson diversity, Richness, pH, SOC, TN (*P* < 0.05). There were significantly correlations between C/P and Shannon-Weiner, Richness, pH, SOC, TN (*P* < 0.05). There were significantly correlations between N/P and Shannon-Weiner, Richness, pH, SOC, TN (*P* < 0.05). There were significantly correlations between Alkaline phosphatase and total coverage, Shannon-Weiner, Simpson diversity, Richness, pH, SOC, TN (*P* < 0.05). There was significantly correlations between Catalase and total coverage (*P* < 0.05). RDA analysis was carried out on the environmental factors and soil soil stoichiometric, enzyme activity in Fig 4. As shown Fig 4, in the first two axes of environmental factors explained 99.97% (Fig 4A) and 99.87% (Fig 4B) of soil stoichiometric and enzyme activity, which had biological statistical significance. That was, the first two axes can more completely reflect the information of soil stoichiometric and enzyme activity with environmental factors. Based on the Monte Carlo test in the RDA analysis (Table 4), Richness index, SOC and TN significantly affected soil stoichiometric (P < 0.05), while Simpson diversity index and pH significantly affected soil activity (P < 0.05). That was, pH, TN, SOC, Richness index and Simpson diversity index were the main environmental factors affecting the soil C:N:P stoichiometry and enzyme activities in different grassland types in Qilian Mountain nature reserve. Cluster analysis based on vegetation and soil variables shows that 7 grassland types are clustered into three categories (Fig 5). The first category was UM, AS, AM, TS, the second category was TDS and TD, and the third category was AD.

**Table 3:** Correlation analysis.

Correlation	C/N	C/P	N/P	Urease	Alkaline phosphatase	Catalase	Sucrase
Total Coverage	0.802*	0.665	0.573	0.606	.775*	0.788*	0.624
Grass layer height	-0.283	-0.39	-0.547	-0.25	-0.633	0.124	-0.625
AGB	0.59	0.591	0.441	0.299	0.421	0.67	0.147
Shannon-Weiner	0.909**	0.817*	.755*	0.659	0.920**	0.71	0.714
Pielou evenness	0.194	-0.017	0.078	0.34	0.389	-0.169	0.554
Simpson diversity	-0.895**	-0.747	-0.727	-0.752	-0.966**	-0.669	-0.850*
Richness	0.820*	0.884**	0.815*	0.474	0.778*	0.668	0.472
pH	-0.759*	-0.834*	-0.868*	-0.486	-0.931**	-0.311	-0.696
SOC	0.905**	0.931**	0.824*	0.165	0.744	0.645	0.383
TN	0.869*	0.925**	0.869*	0.242	0.796*	0.624	0.486
TP	0.519	0.274	0.145	0.237	0.551	0.329	0.455

**Table 4:** Monte Carlo test at the soil stoichiometric and enzyme activity with environmental factors.

Name	soil C:N:P stoichiometry			Name	Soil enzyme activities		
	Contribution %	pseudo-F	P		Contribution %	pseudo-F	P
SOC	86.4	31.8	**0.006**	Simpson diversity index	90.7	48.5	**0.002**
TP	9.5	9.2	**0.044**	pH	5.9	6.8	**0.046**
Richness index	2.8	6.4	**0.036**	TN	2.6	8.6	0.072
pH	0.9	5	0.072	TP	0.6	3.2	0.18
Pielou Evenness index	0.4	14.4	0.244	Grass layer height	0.2	2	0.365
Grass layer height	<0.1	<0.1	1	SOC	0.1	<0.1	1

## Discussion

Soil ecological stoichiometric characteristics (C/N, C/P, and N/P) have a strong regulatory effect on the carbon fixation process in terrestrial ecosystems (Zhang et al., 2016) [32], which is an important parameter to measure soil quality [33], reflecting the ability of soil to release nitrogen and mineralized phosphate nutrients. Due to the influence of climate, landform, soil biology, and human interference, the total amount of soil carbon, nitrogen, and phosphorus varies greatly [34,35]. Among them, C/N is an indicator of the decomposition speed of soil organic matter, which affects the internal circulation of soil C and N elements, and is inversely proportional to the rate of organic matter decomposition [36]. C/P is a reflection of the P release and P sequestration potential of soil decomposing organic matter [37,38]. N/P is an indicator of the abundance and deficiency of soil nutrient supply [39]. In ours research, C/N of different grasslands ranged from 5.08 to 17.35, C/P of different grasslands ranged from 2.50 to 72.29, and N/P of different grasslands ranged from 0.53 to 4.04. That was, the overall organic matter decomposition in Qilian Mountains grasslands was slow and the mineralized decomposition P capacity was limited, and the plants’ productivity of different grassland was mainly limited by nitrogen. In ours research, there were significant differences in C/N, C/P, and N/P among different grassland types, and the water and heat conditions in the distribution areas of different grassland types were different, which was the main reason for the differences in C/N, C/P, and N/P. At the same time, since C and N almost simultaneously respond to environmental changes, this also indirectly reflects the principle of stoichiometry, that is, C and N are structural components, and the organic matter accumulation formation and digestion require relative amounts of N, fixed amount of C and other nutrients.

Soil enzymes can decompose complex organic compounds into smaller organic compounds and inorganic nutrients. Soil enzymes are the most active and sensitive components in soil, promoting the nutrient cycle of soil and the supply of nutrients needed for plant growth [40]. Soil enzymes are an important index for evaluating the soil quality of different grasslands [41,42]. In ours research, there were significant differences in alkaline phosphatase, urease, sucrase, and catalase among different grassland types.Due to differences in the distribution area and plant composition of different grasslands, the characteristics of vegetation community structure (height, coverage) are different, leading to heterogeneity in the absorption of light and heat resources, further causing differences in hydrothermal conditions and aeration conditions in soil [4,43]. Besides, the difference in growth status and litter of different grasslands affects soil microbial biomass and flora composition, thus leading to differences in soil enzyme activity [31].

There are interactions between soil stoichiometric characteristics, soil enzyme activities and environmental factors. And vegetation characteristics, and soil indicators of grassland are the most intuitive forms to characterize the attributes and characteristics of grassland [19,20]. In ours research, there were significantly correlations between C/N and total coverage, Shannon-Weiner, Simpson diversity, Richness, pH, SOC, TN, and significantly correlations between C/P and Shannon-Weiner, Richness, pH, SOC, TN, and significantly correlations between N/P and Shannon-Weiner, Richness, pH, SOC, TN, which was reflecting the coupling relationship between soil stoichiometric characteristics and environmental factors. Meanwhile, RDA analysis found that, Richness index, SOC and TN significantly affected soil stoichiometric, which was similar to the findings of Deng et al [44]. Abiotic factors can indirectly affect soil enzyme activity by altering soil microbial activity or community structure [12], while soil nutrient cycling and carbon turnover depend on soil enzyme activity [45]. The key factors affecting soil enzyme activity (SOM content, N:P, total nitrogen content, number of bacteria, number of fungi, number of actinomycetes) include both biological and abiotic factors. In ours research, there were significantly correlations between Alkaline phosphatase and total coverage, Shannon-Weiner, Simpson diversity, Richness, pH, SOC, TN, and significantly correlations between Catalase and total coverage. Meanwhile, RDA analysis found that, Simpson diversity index and pH significantly affected soil activity, which was similar to the findings of Wang et al. [46]. The seven grassland types were clustered into three categories (AD, TDS and TD, others types grassland). That was, different types of grasslands in the Qilian Mountains can be divided into three groups for ecological management, effectively solving the problems caused by the large differences in uses and functions of different grasslands and the distribution of small patches to the management.

## Conclusion

This study has demonstrated that stoichiometric characteristics and soil enzyme activities of different grasslands vary with grassland types. Based on N/P ratio and RDA analysis, nitrogen was the main factor limiting the grassland productivity, and pH, TN, SOC, Richness index and Simpson diversity index were the main environmental factors affecting the soil C:N:P stoichiometry and enzyme activities. Cluster analysis showed that 7 grassland types were clustered into three categories, that was, the grassland Qilian Mountain can be managed in the ecological district basing in three categories of clustering results. The results of this study can provide data support and theoretical guidance for the scientific management and ecological protection of grassland in Qilian Mountains Reserve.

## Supporting information

S1 Data (RAR)
